# Papers Read before Medical and Dental Societies

**Published:** 1880-03

**Authors:** 


Papers Read before Medical and Dental Societies, and the Proceedings of such Societies.—We are always glad to receive and to publish the carefully prepared papers that are read before medical societies, as we are generally pretty certain of getting something in them that will interest and often instruct our readers; and the brief, voluntary papers, too, that are sometimes read before such bodies are frequently of much intrinsic value, and would receive careful attention at our hands. But we do not care, as a general rule, to publish the debates or dissensions on such papers, as they occur very often before societies. Those dissensions are occasionally, though fortunately not always, prompted by a feeling that "despises the excellence it cannot reach;" or, else, the comments are, in fulsome panegyric of the author, or production, by another class of disputants. In other instances, again, the comments are by persons who neither appreciate, nor care, if they comprehend the merits of the production, and seem regardless of the feelings of the author so long as they know their names will be found among the disputants. They well know that it is easier to criticise than to prepare a paper for such an occasion, but they, doubtless, care far more for seeing their names in print than for any other motive, and "to this extent, no more," their comments or criticisms are introduced. In any case, even when comments are made by fair-minded and intelligent men, the "snap judgment" or hasty conclusion on the merits of papers of this description is rarely of much or permanent value to the profession. Hence, we have not thought of devoting our space to the publication of such proceedings. We prefer well digested and carefully prepared articles and ascertained facts, to any running comments from Drs. S——, B—— and J—— on the paper they have heard. In fact, we are not in favor of medical debating societies, wherein members voluntarily array themselves on the positive and negative sides of a carefully prepared essay, which for obvious reasons could be better appreciated at home. The little advertising of themselves and their specialties, where they profess them, would seem, from some reports we read, to be the leading motives that actuate such men in speaking on these occasions. Cheap Johnism should be deprecated in medicine as elsewhere; for no mattter how skillfully and artistically the wares may be displayed, the proprietor is always found behind the counter ready to perform his traditional work. We are far from opposed to medical societies debating professional topics, *pro* and *con*; but to do so profitably to themselves and others, the subject or subjects for discussion should be known and studied in advance of the time of meeting. Precipitate judgment and hasty conclusions on subjects so closely related to our physical, and sometimes our moral well-being, also, as often find legitimate field for investigation and discussion in our medical societies and associations, should not be allowed to make their way into print if they must occur at all. It is the abuse of a privilege, and not the use, to which attention is attempted to be called.

